# Effectiveness and cost-effectiveness analysis of nimotuzumab for the radiotherapy of locoregionally advanced nasopharyngeal carcinoma

**DOI:** 10.1186/s13014-020-01674-5

**Published:** 2020-10-02

**Authors:** Zhaodong Fei, Ting Xu, Mengying Li, Taojun Chen, Li Li, Xiufang Qiu, Chuanben Chen

**Affiliations:** 1 Department of Radiotherapy, Fujian Medical University Cancer Hospital and Fujian Cancer Hospital, Fujian Medical University, Fuzhou, People’s Republic of China; 2 Fujian Medical University, Fuzhou, People’s Republic of China; 3 Department of Radiation Oncology, Fujian Medical University Cancer Hospital and Fujian Cancer Hospital, Fuma Road, Fuzhou, 350014 Fujian People’s Republic of China

**Keywords:** Nasopharyngeal carcinoma, Nimotuzumab, Cost-effectiveness analysis, Intensity-modulated radiotherapy, Concurrent chemoradiotherapy

## Abstract

**Background:**

This study aimed to assess the effectiveness and cost-effectiveness of nimotuzumab in patients with locoregionally advanced nasopharyngeal carcinoma (LA-NPC).

**Methods:**

LA-NPC patients treated between October 2013 and December 2016 were retrospectively reviewed. A well-balanced cohort of patients who received nimotuzumab in addition to standard treatment (n = 50) and patients who did not receive nimotuzumab (n = 100) was selected using propensity score-matching method (1:2 ratio) for the cost-effectiveness analysis.

**Results:**

Compared with concurrent chemoradiotherapy (CCRT) alone, addition of nimotuzumab to CCRT significantly improved the 3-year overall survival (OS) (98.00% vs. 91.00%, *P* = 0.032). On multivariate analysis, nimotuzumab (hazard ratio = 0.124, 95% confidence interval: 0.017–0.902, *P* = 0.039) showed prognostic significance for OS. No serious treatment-related adverse events were observed in the nimotuzumab group (*P* > 0.05). Cost-effectiveness analysis revealed that addition of nimotuzumab increased the average treatment costs by $14,364.63. The additional cost for every one percent increase in OS rate was $ 2,052.09.

**Conclusion:**

Addition of nimotuzumab to CCRT for LA-NPC confers significant survival benefits; however, it is not cost-effective.

## Introduction

Nasopharyngeal carcinoma (NPC) is a highly aggressive malignant tumor derived from nasopharyngeal epithelial cells. The disease is endemic in South China, Southeastern Asia, Middle East and North Africa [1, 2]. For anatomic constrain, up to 70% of newly diagnosed patients with nasopharyngeal cancer have locoregionally advanced disease [3] with an unfavorable prognosis. Platinum-based concurrent chemoradiotherapy (CCRT) with or without induction chemotherapy (IC) is the standard treatment for patients with locoregionally advanced nasopharyngeal carcinoma (LA-NPC) [4]. Application of intensity-modulated radiotherapy (IMRT) has helped improved locoregional control and survival [5, 6]. However, approximately 20% of patients with locoregionally advanced disease tend to develop recurrent disease and/or metastasis [7]. Therefore, optimizing the systemic treatment strategies is a key imperative to improve the survival of patients with locoregionally advanced disease.

Epidermal growth factor receptor (EGFR) overexpression is observed in more than 80% of NPC [8], and is associated with tumor invasiveness, treatment resistance, and poor prognosis [9]. Nimotuzumab (NTZ) is a blocking monoclonal antibody against EGFR, which is highly humanized and has a higher effective dose concentration compared with other anti-EGFR drugs [10]. Nimotuzumab is designed to reduce immunoreactivity and to enhance radio sensitivity with few serious complications and acceptable safety. Furthermore, several studies have demonstrated the favorable efficacy and safety profile of nimotuzumab administered in combination with CCRT in patients with LA-NPC [11]. In a multicenter randomized controlled study, induction therapy administered in combination with nimotuzumab showed better response than chemotherapy alone (77.8% vs 63.0%, *P* = 0.033) in patients with stage III-IVa NPC with EGFR expression [12]. Currently, nimotuzumab is approved only for the treatment of EGFR-positive stage III–IV NPC in combination with radiotherapy or chemoradiotherapy.

NPC has a high incidence in southeast China’s Fujian province (population 39.41 million). Our cancer center is the only specialist cancer hospital in Fujian. NPC is the dominant disease in our hospital, and it is representative in the epidemic area. The high cost of nimotuzumab has prevented its wider use for the treatment of NPC. The cost-effectiveness of addition of nimotuzumab to standard treatment for NPC is not known. In this study, the propensity score-matching (PSM) method was used to retrospectively analyze the efficacy of nimotuzumab in LA-NPC. Based on the analyses, we assessed the cost-effectiveness of nimotuzumab in combination with standard treatment for LA-NPC.

## Materials and methods

### Patients

We reviewed the medical records of 1753 consecutive patients with newly confirmed NPC who under-went complete treatment at our center between October 2013 and December 2016. The inclusion criteria were: (1) stage III-IV disease in accordance to the American Joint Committee on Cancer (AJCC) staging system (7th edition, 2010); (2) confirmed by pathology; (3) positive EGFR expression; (4) Karnofsky performance score (KPS) ≥ 80; (5) treatment regimen: IC followed by CCRT with or without nimotuzumab; (6) radical IMRT. The exclusion criteria were as follows: (1) diagnosed with a previous malignancy or other concomitant malignant disease; (2) pregnancy or lactation; (3) received other anti-EGFR targeting therapy; (4) metastatic disease at diagnosis; (5) patients > 70 years old. Based on these criteria, 394 patients were selected for the matched study.

### Treatment

#### Chemotherapy

IC followed by CCRT was recommended for patients with stage III–IV disease at our institution during the study reference period. The chemotherapy regimen comprised of 2–3 cycles of IC prior to CCRT. The IC regimen consisted of gemcitabine (1000 mg/m^2^, on days 1 and 8) plus cisplatin (80 mg/m^2^, on day 2); or paclitaxel (135 mg/m^2^, on day 1) plus cisplatin (80 mg/m^2^, on day 2). CCRT based on cisplatin (80 mg/m^2^) was administered intravenously for 2 cycles until the completion of RT. Chemotherapy, including CCRT and IC, were repeated every 3 weeks. Radiotherapy was administered simultaneously with the first cycle of concurrent chemotherapy [13].

#### Radiotherapy

All patients were treated with Volumetric Modulated Arc Therapy (VMAT), which is a novel form of IMRT. The target volume and radiotherapy dose were delineated using an institutional treatment protocol as previously reported [14]. The primary gross tumor volume (GTV-P) and the cervical metastatic lymph nodes (GTV-N), included all gross disease was determined by imaging (CT and MRI fusion), clinical, and endoscopic results. The dose to the GTV-P and GTV-N was 69.7–70.0 Gy administered in 31–35 fractions; the corresponding dose to the high-risk region (CTV1) was 62–62.7 Gy, while dose to the subclinical prophylactic low-risk region (CTV2) was 54.4–56.2 Gy administered in the same number of fractions. The planning target volume (PTV) was created on the basis of each volume with an additional 3 mm margin. Organ at risk (OAR) include the brain stem, spinal cord, optic nerve, optic chiasm, temporal lobe, crystal, and parotid, pituitary and mandibular glands.

#### Nimotuzumab

Owing to the high cost of nimotuzumab, its use depended on the patient’s preference, affordability, and the physician’s experience. Nimotuzumab was administered concomitantly with radiotherapy at a dose of 200 mg once weekly for 8 weeks, commencing on a day before IMRT. A total of 50 patients received full doses of nimotuzumab.

### Follow-up and clinical endpoints

After completing standard treatment, the therapeutic effects were evaluated every 3 months for the first 2 years, then every 6 months up to the first 5 years, and once a year thereafter. Nasopharyngoscopy, enhanced MRI of the head and neck, chest computed tomography (CT), and abdominal ultrasound were routinely performed. In consideration of the value of 18F-Fluorodeoxyglucose positron emission tomography and CT (PET-CT) in the diagnosis and treatment of nasopharyngeal cancer, the PET-CT should be performed when the physician considers it necessary [15]. The final date of follow-up was January 2020.

The primary clinical endpoints were overall survival (OS, defined as time from diagnosis to death for any cause) and progression-free survival (PFS, time from diagnosis to disease progression or death from any cause), other outcome variables included locoregional relapse-free survival (LRRFS, time from diagnosis to local or regional recurrence or both) and distant metastasis-free survival (DMFS, time from diagnosis to first distant metastasis). The duration was calculated from the date of diagnosis to the date of each event or last follow-up. Acute toxicity was graded according to the Common Terminology Criteria for Adverse Events (version 3.0).

### Statistical analysis

**Fig. 1:**
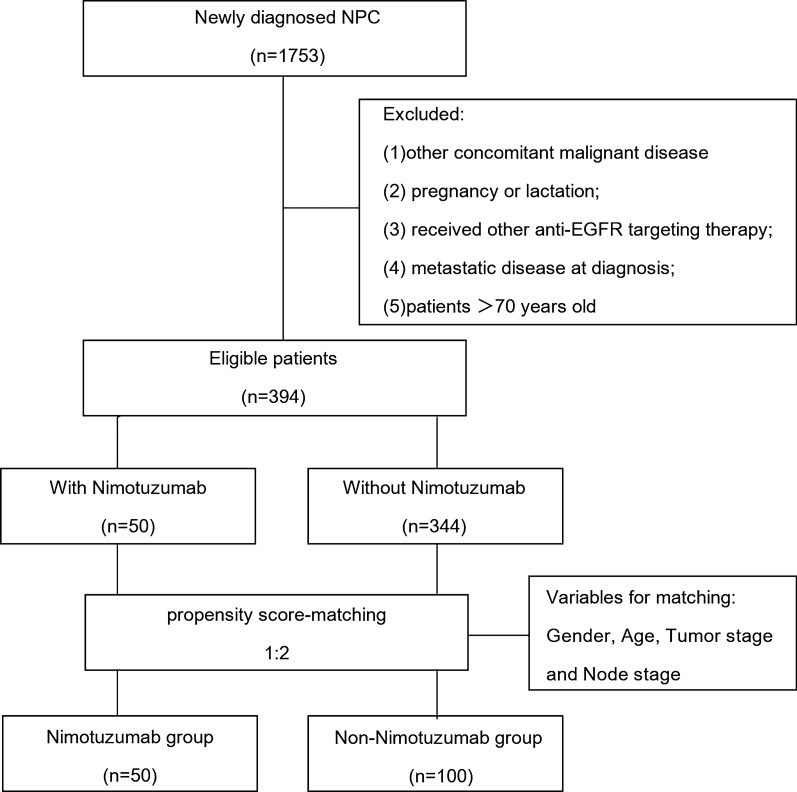
Study flow diagram

The baseline demographic and clinical characteristics of the study population were summarized (Table 1) and the differences between the nimotuzumab group and non-nimotuzumab group were compared using the Chi-squared test. Logistic regression analysis was used to identify confounders between the treatment groups. Propensity score-matching method was used in a 1:2 ratio to balance various factors, including sex, age, tumor stage (T stage), and node stage (N stage). Using a caliper width of 0.1, 1:2 matching was performed between patients in the nimotuzumab group and non-nimotuzumab group based on the propensity scores. After propensity score matching, a total of 150 patients were selected (50 patients in the nimotuzumab arm and 100 patients in the non-nimotuzumab arm) (Fig. 1). OS, PFS, LRRFS, and DMFS were calculated using the Kaplan–Meier method. Between-group differences in survival outcomes were assessed using the log-rank test. Multivariate analysis was performed using the Cox proportional hazards models. All statistical analyses were performed using the SPSS 22.0 (IBM Corporation, Chicago, IL, USA) and R 2.15.3 software. All tests were two-tailed and *P* values < 0.05 were considered indicative of statistical significance.

**Table 1:** Patient baseline characteristics of NPC patients before PSM

Characteristic	NTZ group N = 50 (%)	Non-NTZ group N = 344 (%)	*P* value
Gender			0.870
Male	34 (68.00)	240 (69.77)	
Female	16 (32.00)	104 (30.23)	
Age			0.387
≤ 50	33 (66.00)	205 (59.59)	
> 50	17 (34.00)	139 (40.41)	
T classification			0.763
T1	6 (12.00)	34 (9.88)	
T2	7 (14.00)	50 (14.53)	
T3	20 (40.00)	138 (40.17)	
T4	17 (34.00)	122 (35.47)	
N classification			0.855
N0	3 (6.00)	30 (8.72)	
N1	17 (34.00)	111 (32.27)	
N2	18 (36.00)	119 (34.59)	
N3	12 (24.00)	84 (24.42)	
Clinical stage			0.840
III	23 (46.00)	153 (44.48)	
IV	27 (54.00)	191 (55.52)

Based on the retrospectively analysis using PSM method, we performed a cost-effectiveness analysis from the perspective of traditional payers to measure the therapeutic value of nimotuzumab in LA-NPC [16, 17]. In this study, the direct costs in each patient’s statement of accounts during the in-hospital period were calculated as total costs, irrespective of the mode of payment (insurance or no insurance). The total costs include cost of anti-tumor drugs, radiotherapy, supportive drugs, hospitalization, therapies related to 3–4 AEs, and imaging or biochemical investigations [18, 19]. Indirect and implicit costs were ignored as a consequence of individual differences [20]. All costs are expressed in U.S. Dollars (USD), and the exchange rate in December 2016 was used [1 USD = 6.88 China Yuan (CNY)] [21].

The 3-year OS and 3-year PFS rates were used as measures of effectiveness. Cost-effectiveness ratio (C/E%) and the incremental cost-effectiveness ratio (ICER) were used as outcome measures for the cost-effectiveness analysis. The ICER was calculated by dividing the total cost difference between the nimotuzumab group and non-nimotuzumab group by the difference in effectiveness between the two groups [21]. In view of the recommendations of the 2015 China Guidelines for Pharmacoeconomic Evaluations and Manual, both the costs and the utility values were discounted at an annual rate of 3% [22].

## Results

### Patient characteristics

Clinical data pertaining to a total of 1753 consecutive NPC patients treated with IMRT were reviewed. Finally, 394 patients were eligible for propensity score-matching (Fig. 1). Out of the entire cohort of 394 LA-NPC patients, 50 patients treated with nimotuzumab were categorized as nimotuzumab group and 344 patients who did not receive nimotuzumab were categorized as non-nimotuzumab group. The basic characteristics of all patients are summarized in Table 1. To generate a comparable non-nimotuzumab group in a ratio of 1:2, 100 patients were selected by PSM from 344 patients. Gender, age, T stage, and N stage were used as matching factors and patient characteristics were well balanced between the two propensity-matched groups. Ultimately, nimotuzumab group included 50 patients who had received 2–3 cycles of IC followed by CCRT with nimotuzumab, and non-nimotuzumab group included 100 patients who had received 2–3 cycles of IC followed by CCRT without nimotuzumab. The baseline characteristics of PSM cohorts are summarized in Table 2.

**Table 2:** Patient baseline characteristics of NPC patients after PSM

Characteristic	NTZ group N = 50 (%)	Non-NTZ group N = 50 (%)	*P* value
Gender			0.856
Male	34 (68.00)	66 (66.00)	
Female	16 (32.00)	34 (34.00)	
Age			1.00
≤ 50	33 (66.00)	66 (66.00)	
> 50	17 (34.00)	34 (34.00)	
T classification			0.937
T1	6 (12.00)	11 (11.00)	
T2	7 (14.00)	10 (10.00.)	
T3	20 (40.00)	48 (48.00)	
T4	17 (34.00)	31 (31.00)	
N classification			0.925
N0	3 (6.00)	9 (9.00)	
N1	17 (34.00)	28 (28.00)	
N2	18 (36.00)	42 (42.00)	
N3	12 (24.00)	21 (21.00)	
Clinical stage			0.565
III	23 (46.00)	51 (51.00)	
IV	27 (54.00)	49 (49.00)	

In the entire cohort of 394 patients, the median age of patients was 47 (range 17–70) years, the ratio of male (n = 274) to female (n = 120) was 2.28:1, and the median duration of follow-up was 45 months (range 5–73).

### Survival outcomes and effectiveness

**Fig. 2:**
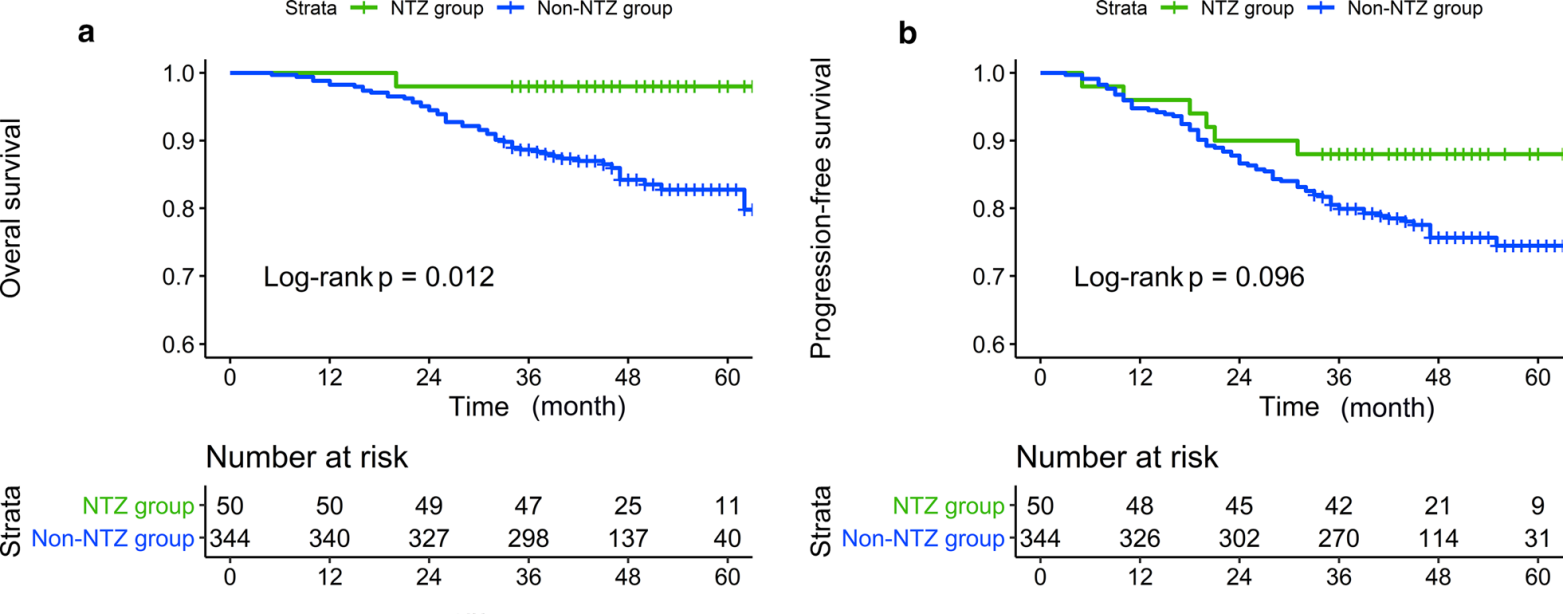
The survival curves of OS and PFS for nimotuzumab group versus non-nimotuzumab group before PSM

In the original unmatched cohort of 394 patients, the 3-year OS, PFS, LRRFS and DMFS rates for nimotuzumab group vs. non-nimotuzumab group were 98.00% versus 88.66% (*P* = 0.013), 88.00% versus 79.94% (*P* = 0.099), 96.00% versus 95.06% (*P* = 0.525), and 92.00% versus 89.24% (*P* = 0.469), respectively. Significant difference in OS was observed between the two groups. The survival curves for OS and PFS are shown in Fig. 2.

**Fig. 3:**
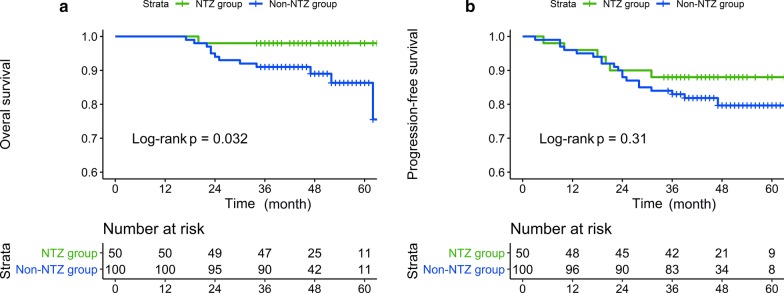
The survival curves of OS and PFS for nimotuzumab group versus non-nimotuzumab group after PSM

In the propensity-matched cohort of 150 patients, the 3-year OS, PFS, LRRFS and DMFS rates for nimotuzumab group versus non-nimotuzumab group were 98.00% versus 91.00% (*P* = 0.032), 88.00% versus 83.00% (*P* = 0.306), 96.00% versus 93.00% (*P* = 0.444) and 92.00% versus 93.00% (*P* = 0.991.), respectively. Significant difference in OS was observed between the two groups. The survival curves for OS and PFS are shown in Fig. 3.

### Multivariate analysis

The OS and PFS of the 394 eligible patients were analyzed by Cox regression models (Table 3). Based on the results of previous studies and the univariate analysis, multivariate analysis was performed to evaluate the following potential prognostic factors: age, gender, T stage, N stage, clinical stage, and nimotuzumab. The results indicated that nimotuzumab treatment [hazard ratio (HR) = 0.124, 95% confidence interval (CI): 0.017–0.902, *P* = 0.039] had prognostic significance for OS. Higher N stage was an independent predictor of poorer OS and PFS.

**Table 3:** Cox regression model of multivariable analysis for OS and PFS

	OS			PFS		
	HR	95% CI	*P* value	HR	95% CI	*P* value
Gender						
Female	1			1		
Male	1.175	0.658–2.100	0.586	0.907	0.564–1.461	0.689
Age						
≤ 50	1			1		
> 50	1.570	0.906–2.718	0.108	1.110	0.715–1.725	0.641
T stage						
T1	1			1		
T2	1.496	0.450–4.979	0.511	1.096	0.424–2.835	0.850
T3	1.564	0.504–4.853	0.439	1.294	0.541–3.095	0.562
T4	3.239	0.870–12.056	0.080	4.012	1.462–11.007	0.007
N stage						
N0	1			1		
N1	4.041	0.533–30.616	0.176	2.162	0.650–7.190	0.208
N2	5.758	0.762–43.511	0.090	3.081	0.931–10.199	0.065
N3	10.354	1.219–87.914	0.032	7.165	1.924–26.678	0.003
Nimotuzumab						
Without	1			1		
With	0.124	0.017–0.902	0.039	0.511	0.223–1.173	0.113

### Cost-effectiveness analysis and sensitivity analysis

After propensity score-matching, the total costs of nimotuzumab group (n = 50) and non-nimotuzumab group (n = 100) were $ 34,135.80 and $ 19,771.17, respectively. Addition of nimotuzumab increased the treatment costs by $14,364.63. Thus, the cost-effectiveness ratio (C/E%) of 3-year OS and 3-year PFS in nimotuzumab group vs. non-nimotuzumab group were $ 348.32 versus $ 217.27 and $ 387.91 versus $ 238.21, respectively. The difference of effectiveness (3-year OS) was 7%. Therefore, the ICER was calculated as $ 2052.09, which means that every one percent increase in overall survival rate by using nimotuzumab costed an additional $ 2052.09.

We varied overall survival and progression-free survival by ± 10% in the sensitivity analyses; thus, the C/E% of 3-year OS and 3-year PFS in nimotuzumab group versus non-nimotuzumab group were $ 387.91 versus $ 244.09 and $ 437.64 versus $ 270.84, respectively.

### Adverse reactions

Table 4 showed the incidence of acute toxicity in 394 patients. Hematological toxicity was the most frequently observed adverse reaction in the Nimotuzumab group. Nevertheless, there were no significant differences between the two groups with respect to hematologic parameters (*P* > 0.05). There was no significant between-group difference with respect to hepatoxicity, nephrotoxicity, gastrointestinal reactions, or acute radiation dermatitis and mucositis (*P* > 0.05 for all). Overall, treatment toxicity was well-tolerated, and no treatment-related deaths occurred in either group.

**Table 4:** Acute toxicities in the 394 NPC patients

Acute toxicity	NTZ group N = 50 (%)	Non-NTZ group N = 344 (%)	*P* value
Leukopenia			0.739
G0–G1	18 (36.00)	129 (37.50)	
G2	14 (28.00)	100 (29.07)	
G3	14 (28.00)	91 (26.45)	
G4	4 (8.00)	24 (6.98)	
Neutropenia			0.947
G0–G1	26 (52.00)	173 (50.29)	
G2	13 (26.00)	98 (28.49)	
G3	10 (20.00)	73 (21.22)	
G4	1 (2.00)	0	
Anemia			0.811
G0–G1	40 (80.00)	279 (81.10)	
G2	7 (14.00)	51 (14.83)	
G3	3 (6.00)	14 (4.07)	
Thrombocytopenia			0.650
G0–G1	45 (90.00)	316 (91.86)	
G2	4 (8.00)	24 (6.98)	
G3	1 (2.00)	4 (1.17)	
Hepatotoxicity			0.760
G0–G1	43 (86.00)	290 (84.30)	
G2	5 (10.00)	39 (11.34)	
G3	2 (4.00)	15 (4.36)	
Nephrotoxicity			0.671
G0–G1	47 (94.00)	318 (92.44)	
G2	3 (6.00)	19 (5.52)	
G3	0	7 (2.03)	
Skin reaction			0.866
G0–G1	37 (74.00)	257 (74.71)	
G2	11 (22.00)	79 (22.97)	
G3	2 (4.00)	8 (2.33)	
Mucositis			0.594
G0–G1	16 (32.00)	117 (34.01)	
G2	19 (38.00)	139 (40.41)	
G3	13 (26.00)	76 (22.09)	
G4	2 (4.00)	12 (3.49)	
Nausea			0.895
G0–G1	27 (54.00)	178 (51.74)	
G2	17 (34.00)	130 (37.79)	
G3	4 (8.00)	31 (9.01)	
G4	2 (4.00)	5 (1.45)	
Vomiting			0.803
G0–G1	35 (70.00)	233 (67.73)	
G2	9 (18.00)	72 (20.93)	
G3	6 (12.00)	39 (11.34)	
Diarrhea			0.693
G0–G1	44 (88.00)	309 (89.83)	
G2	6 (12.00)	35 (10.17)	
Weight loss			0.623
G0–G1	38 (76.00)	271 (78.78)	
G2	10 (20.00)	65 (18.90)	
G3	2 (4.00)	8 (2.33)	

## Discussion

Radio-chemotherapy is the standard treatment modality for stage III-IV NPC. Even with the best available treatment according to guidelines, approximately 5–15% of patients develop local failure, and 15–30% develop distant failure [23]. To further improve the therapeutic outcomes, many clinical trials have explored the effects of radiotherapy and chemotherapy administered in combination with novel therapies. With in-depth characterization of the molecular mechanisms of carcinogenesis and cancer progression, molecular targeted therapy for NPC patients has become a research hotspot [10]. The high expression of EGFR in NPC has been evaluated as a potential therapeutic target. Activation of EGFR pathway was shown to promote tumor cell growth, invasion and angiogenesis, prevent apoptosis, and induce chemoresistance and radioresistance [24].

Although there is no clear consensus, most studies suggest that anti-EGFR monoclonal antibodies, especially nimotuzumab and cetuximab, confer significant benefits in patients with LA-NPC. According to a meta-analysis, addition of anti-EGFR monoclonal antibodies to standard therapy for NPC significantly improved OS (HR, 0.51; 95% CI, 0.39–0.66) compared to standard therapy alone [25]. In a case–control study based on intelligence platform, concurrent administration of nimotuzumab/cetuximab with IC was found to be more effective, with a significant improvement in 3-year disease-free survival rate (84.3% vs. 74.3% *P* = 0.027) [9].

As the most commonly used anti-EGFR monoclonal antibody, cetuximab has shown good curative effect in the treatment of NPC; however, its use is associated with severe adverse reactions, such as oral mucositis and itchy rash [26]. To minimize the toxicity, a drug with a lower affinity constant, nimotuzumab, was developed; nimotuzumab shows a high uptake by tumor and low uptake by normal tissues [27]. Nimotuzumab selectively binds to tumors with moderate to high EGFR expression and rarely causes severe adverse reactions of skin and mucosa. Besides, it displays a longer half-life and elevated area under the curve than cetuximab at equivalent doses [28]. Many clinical trials have demonstrated that concomitant administration of nimotuzumab with concurrent radiotherapy may facilitate radiosensitivity and thus increase treatment efficacy [12, 29, 30]. A phase II clinical study of IC and sequential nimotuzumab combined with CCRT for NPC in stage N3 yielded a satisfactory survival benefit and tolerable toxicity, with 3-year OS, DMFS, and PFS rates of 85.6, 81.9, and 79.5%, respectively [29]. A retrospective paired analysis found that, compared to CCRT alone, CCRT plus nimotuzumab significantly improved the 5-year OS (96.8% vs. 82.3%; *P* = 0.001), DMFS (90.3% vs. 80.6%, *P* = 0.012), and PFS (83.9% vs. 71.0%, *P* = 0.006) rates [30]. These findings indicate a synergistic effect of nimotuzumab and radiotherapy in NPC.

The current study retrospectively analyzed the therapeutic efficacy in 394 patients with stage III-IV EGFR-positive NPC who received standard treatment with or without nimotuzumab. Consistent with previous studies, addition of nimotuzumab to standard treatment was shown to confer significant survival benefit and tolerable adverse reactions for LA-NPC. In the propensity-matched nimotuzumab group, the 3-year OS was 98.00%. The 3-year OS rate in the nimotuzumab group was significantly greater than that in the non-nimotuzumab group (98.00% vs. 91.00%, *P* = 0.032). On multivariate analysis, nimotuzumab was a significant prognostic factor for OS.

We also assessed the cost-effectiveness of the survival benefits conferred by nimotuzumab in the matched cohort. The average treatment cost in the nimotuzumab group was higher than that in the non-nimotuzumab group by $14,364.63. The C/E% of 3-year OS in nimotuzumab group and non-nimotuzumab group were $ 348.32 and $ 217.27, respectively. The ICER was calculated as $ 2052.09. The results of sensitivity analysis of 3-year OS and 3-year PFS were consistent with this finding. This implies that, although nimotuzumab can confer significant survival benefit, its addition to the current standard treatment for LA-NPC patients is unlikely to be considered as cost effective given the commonly accepted thresholds for cancer drugs in China. Based on the cost-effectiveness analysis, we recommend screening of high-risk patients to receive targeted therapy of nimotuzumab. Nimotuzumab may help improve outcomes of high-risk NPC patients. The high-risk factors of NPC include N2-3 stage, large primary tumor volume, unsatisfactory tumor response after IC, and high Epstein Barr virus DNA level after IC [31].

To the best of our knowledge, this is the first study that evaluated the cost-effectiveness of adding nimotuzumab to standard treatment for stage III-IV EGFR-positive NPC. A key strength of this study was the use of propensity score-matching method to minimize the influence of confounding factors; this allowed for a more robust cost-effectiveness analysis. Moreover, by refining costs and benefits, this analysis provides a comprehensive cost and benefit assessment for clinicians developing treatment plans. However, we acknowledge some limitations in this research. First, as a single-center study, due caution should be exercised while interpreting and extrapolating the results to other populations. Second, due to the small number of cases included in this study, larger studies are required to provide more definitive evidence.

## Conclusion

Addition of nimotuzumab to the current standard treatment for LA-NPC was found to confer significant survival benefits; however, it was not found to be cost effective. Thus, targeted therapy with nimotuzumab should be recommended only for high-risk patients. Clinical trials with sufficiently large patient cohorts are required to confirm the efficacy, feasibility, and cost-effectiveness of nimotuzumab.

## Data Availability

The data sets used and/or analyzed during the current study are available from the corresponding author on reasonable request.

## Abbreviations

LA-NPC: Locoregionally advanced nasopharyngeal carcinoma
CCRT: Concurrent chemoradiotherapy
IC: Induction chemotherapy
IMRT: Intensity-modulated radiotherapy
EGFR: Epidermal growth factor receptor
NTZ: Nimotuzumab
PSM: Propensity score-matching
AJCC: American Joint Committee on Cancer
KPS: Karnofsky performance score
VMAT: Volumetric Modulated Arc Therapy
GTV-P: Primary gross tumor volume
GTV-N: Gross tumor volume of the cervical metastatic lymph nodes
MRI: Magnetic resonance imaging
OAR: Organ at risk
CT: Chest computed tomography
PET-CT: 18F-Fluorodeoxyglucose positron emission tomography and CT
OS: Overall survival
PFS: Progression-free survival
LRRFS: Locoregional relapse-free survival
DMFS: Distant metastasis-free survival
RTOG: Radiation therapy oncology group
USD: U.S. Dollars
CNY: China Yuan
C/E%: Cost-effectiveness ratio
ICER: Incremental cost-effectiveness ratio
